# Influence of contextual socioeconomic position on hypertension risk in low- and middle-income countries: disentangling context from composition

**DOI:** 10.1186/s12889-021-12238-x

**Published:** 2021-12-06

**Authors:** Mustapha S. Abba, Chidozie U. Nduka, Seun Anjorin, Shukri F. Mohamed, Emmanuel Agogo, Olalekan A. Uthman

**Affiliations:** 1 Division of Health Sciences, Warwick Medical School, The University of Warwick, Coventry, CV4 7AL UK; 2 Academic Unit of Primary Care (AUPC) and the NIHR Global Health Research Unit on Improving Health in Slums, University of Warwick, Coventry, UK; 3 Health and Systems for Health Unit, African Population and Health Research Center (APHRC), Nairobi, Kenya; 4 Lown Scholars Program, Department of Global Health and Population, Harvard T.H. Chan School of Public Health, Boston, MA USA; 5 Resolve to Save Lives, Country Office Nigeria, Abuja, Nigeria; 6 Warwick Centre for Global Health Research, The University of Warwick, Coventry, CV4 7AL UK; 7 Division of Epidemiology and Biostatistics, Department of Global Health, Stellenbosch University, Stellenbosch, South Africa; 8 Department of Public Health (IHCAR), Karolinska Institutet, Stockholm, Sweden; 9 Department of Infectious Diseases, Karolinska University Hospital, Stockholm, Sweden

**Keywords:** Hypertension, Blood pressure, Socioeconomic status, Multi-level analysis, Low- and middle-income countries (LMICs), Demographic health survey (DHS)

## Abstract

**Background:**

Hypertension has emerged as the single most significant modifiable risk factor for cardiovascular disease and death worldwide. Resource-limited settings are currently experiencing the epidemiological transition from infectious diseases to chronic non-communicable diseases, primarily due to modifications in diet and lifestyle behaviour. The objective of this study was to examine the influence of individual-, community- and country-level factors associated with hypertension in low- and middle-income countries (LMICs).

**Methods:**

Multivariable multi-level logistic regression analysis was applied using 12 Demographic and Health Survey (DHS) datasets collected between 2011 and 2018 in LMICs. We included 888,925 respondents (Level 1) nested within 33,883 neighbourhoods (Level 2) from 12 LMICs (Level 3).

**Results:**

The prevalence of hypertension ranged from 10.3% in the Kyrgyz Republic to 52.2% in Haiti. After adjusting for the individual-, neighbourhood- and country-level factors, we found respondents living in the least deprived areas were 14% more likely to have hypertension than those from the most deprived areas (OR = 1.14, 95% CI 1.10 to 1.17). We observed a significant variation in the odds of hypertension across the countries and the neighbourhoods. Approximately 26.3 and 47.6% of the variance in the odds of hypertension could be attributed to country- and neighbourhood-level factors, respectively. We also observed that respondents moving to a different neighbourhood or country with a higher risk of hypertension had an increased chance of developing hypertension, the median increase in their odds of hypertension was 2.83-fold (95% CI 2.62 to 3.07) and 4.04- fold (95% CI 3.98 to 4.08), respectively.

**Conclusions:**

This study revealed that individual compositional and contextual measures of socioeconomic status were independently associated with the risk of developing hypertension. Therefore, prevention strategies should be implemented at the individual level and the socioeconomic and contextual levels to reduce the burden of hypertension.

## Introduction

Hypertension is a worldwide public health issue. It is one of the leading causes of morbidity, mortality and disability [9, 23, 27, 37]. The prevalence of hypertension continues to increase in low- and middle-income countries (LMICs), particularly in urban communities [51]. Evidence shows that an individual’s health is determined by the environment in which they live, work, and age [56]. However, due to globalisation and industrialisation, the environment we live in is rapidly changing [51]. This shift has implications on the health and well-being of society, notably the epidemiological transition from infectious to non-communicable diseases (NCDs) in both LMICs and high-income countries (HICs) [39, 47, 56].

Recent research indicates increased salt intake, fatty meals, lack of fruits, physical inactivity, alcohol consumption, smoking, hyperlipidaemia, psychosocial stress, family history, sex, and ageing are common risk factors associated with hypertension [10, 44]. However, there are other emerging risk factors linked to hypertension which include socioeconomic disadvantages such as malnutrition in early childhood, lower income, lower level of education, place of residence, air pollution, employment status, wealth index, comorbidities, etc. [10, 12, 16, 38, 47].

These novel risk factors warrant further exploration, especially in LMICs settings. Harshfield et al. [18], Rahman et al. [42] & Taraque et al. [49]) established that the prevalence and likelihood of developing hypertension vary according to the place of residence. Hypertension is largely untreated in rural settings due to a lack of awareness, limited access to healthcare facilities, and high cost of treatment. Kibra et al. [24] suggest poor sanitation, smoking and alcohol consumption contribute to high blood pressure in urban areas. Socioeconomic factors such as education level, wealth status, and place of residence, among others, influence behavioural risk factors [55]. Urban dwellers consume more calories and have different lifestyle patterns that contribute to obesity, which increases the odds of developing hypertension, according to the National Institute of Population Research and Training (NIPORT) report from 2013 and Rahman et al. [42].

Hypertension research is centred around individual-level socio-demographic issues, even though theories suggest that the distribution and determinants of population health are epistemologically multi-level. Therefore, understanding the determinants of hypertension beyond individual socio-demographic factors is necessary to facilitate appropriate interventions.

The purpose of this study was to examine the influence of individual-, community- and country-level factors associated with hypertension in low- and middle-income countries.

## Methods

### Study design and data

This cross-sectional study data were obtained from Demographic Health Surveys (DHS) from 12 LMIC carried out between 2011 and 2018. Specifically, countries with up-to-date blood pressure measurements were selected. DHS datasets were obtained for Albania, Bangladesh, Benin, Ghana, Haiti, India, the Kyrgyz Republic, Lesotho, Namibia, Nepal, South Africa and Tajikistan. The DHS sample is generally representative of the national residents (urban-rural and regional department states). The sample is based on a stratified 2-stage cluster design. In Stage 1, the enumeration areas were drawn from census files, and in stage 2, in the selected sample from each EA, households are chosen from an updated list. In most countries, the DHS surveys include a household questionnaire, a women’s questionnaire, and a men’s questionnaire. All three questionnaires were implemented across countries with similar implementation protocols, interviewer training and monitoring.

### Outcome variable

Hypertension was defined as an SBP ≥ 140 mmHg or a DBP ≥ 90 mmHg and treatment for hypertension [20]. In DHS surveys, blood pressure was measured with a fully automatic digital blood pressure measuring device that has an automatic upper arm inflation and automatic pressure release [20]. Field staff received training before the survey on the use of the device according to the manufacturers recommended protocol [20]. In most countries, blood pressure was measured three times [20]. The first measurement was discarded, then the average of the last two measurements was reported as the respondent’s blood pressure in millimetres of (mmHg) [53].

### Explanatory variables

#### Individual-level factors

The following individual-level factors were included in the models: sex of the respondent (male or female), age of respondents, level of education (no education, primary, secondary or higher), marital status (never married, currently married or ever married), occupation (working or not working), cigarette smoking (yes or no), health insurance (yes or no), problems getting money needed for treatment (yes or no), media access (radio, television or magazine), and indoor air pollution (cooking fuel type: low-pollution fuel, high-pollution fuel). The weight measurement was obtained to the nearest 0.5 kg with the respondents wearing light clothing. The height was measured to the nearest 0.1 cm. Body mass index (BMI) was categorised into three classes: i) underweight, ii) overweight, and iii) obese, with the following ranges: i) underweight (< 18.5 kg/m^2^), ii) overweight (25–29.9 kg/m^2^), and iii) obese (≥30 kg/m^2^).

The DHS wealth index was used as a proxy indicator to calculate the respondents’ socioeconomic status, as described by Montgomery et al. [36] & Vyas et al. [54] because data on household income and expenditure were not collected in the surveys. Briefly, the wealth index of economic status for each household was constructed using principal component analysis based on the following household variables: number of rooms per house and ownership of a car, motorcycle, bicycle, refrigerator, television, telephone and any kind of heating device. Individual household wealth index scores were calculated by summing the coefficients for the assets or characteristics of each household. The DHS wealth index tertiles (poor, middle, and rich) were calculated and used in the subsequent modelling based on the criteria described above.

#### Neighbourhood-level factors

We defined neighbourhoods as respondents clustering within the same geographical area based on sharing a common primary sample unit within the data. The sampling frame for identifying the primary sample unit in the DHS was drawn from the most recent census. Kravdal [26] & Griffiths et al. [17] suggests that the primary sample unit is the most reliable and suitable identifier of neighbourhood measures across all surveys. Additionally, the sample size per cluster met the optimum size, with a tolerable degree of precision loss across the DHS.

Neighbourhood socioeconomic disadvantage was classified as a community-level variable in this study. We factored in respondents who had no education (illiterate), were unemployed, were rural residents, and were living below the poverty level, especially those with asset index below 20% in the lowest quintile, in estimating the neighbourhood socioeconomic disadvantage. We then generated a standardised score with a mean of 0 and standard deviation of 1 from this index, indicating that greater scores depict a lower socioeconomic position (SEP). To achieve nonlinear effects, we divided the resultant scores into five quintiles to generate easily interpretable results by decision-makers. We derived community-level variables using non-self means or proportions to avoid the overlap of measures between the two levels of analysis. We assigned a value representing the average for all other respondents, excluding those within the cluster.

### Country-level factor

We included the United Nations Development Program (UNDP) ‘s human development index as the country-level factor. The human development index, also known as the intensity of deprivation, is the average percentage of deprivation experienced by people in multidimensional poverty. Like the wealth index, the intensity of deprivation was computed using a principal component based on household deprivation data with regards to education, health and living standards at the country level. We categorised the human development index into three (low, moderate and high) levels [52].

### Statistical analyses

#### Descriptive data analyses

This cross-sectional study performed with a survey and the data obtained were analysed using descriptive statistics. The distribution of respondents stratified by key variables was expressed as percentages.

#### Modelling approaches

**Fig. 1:**
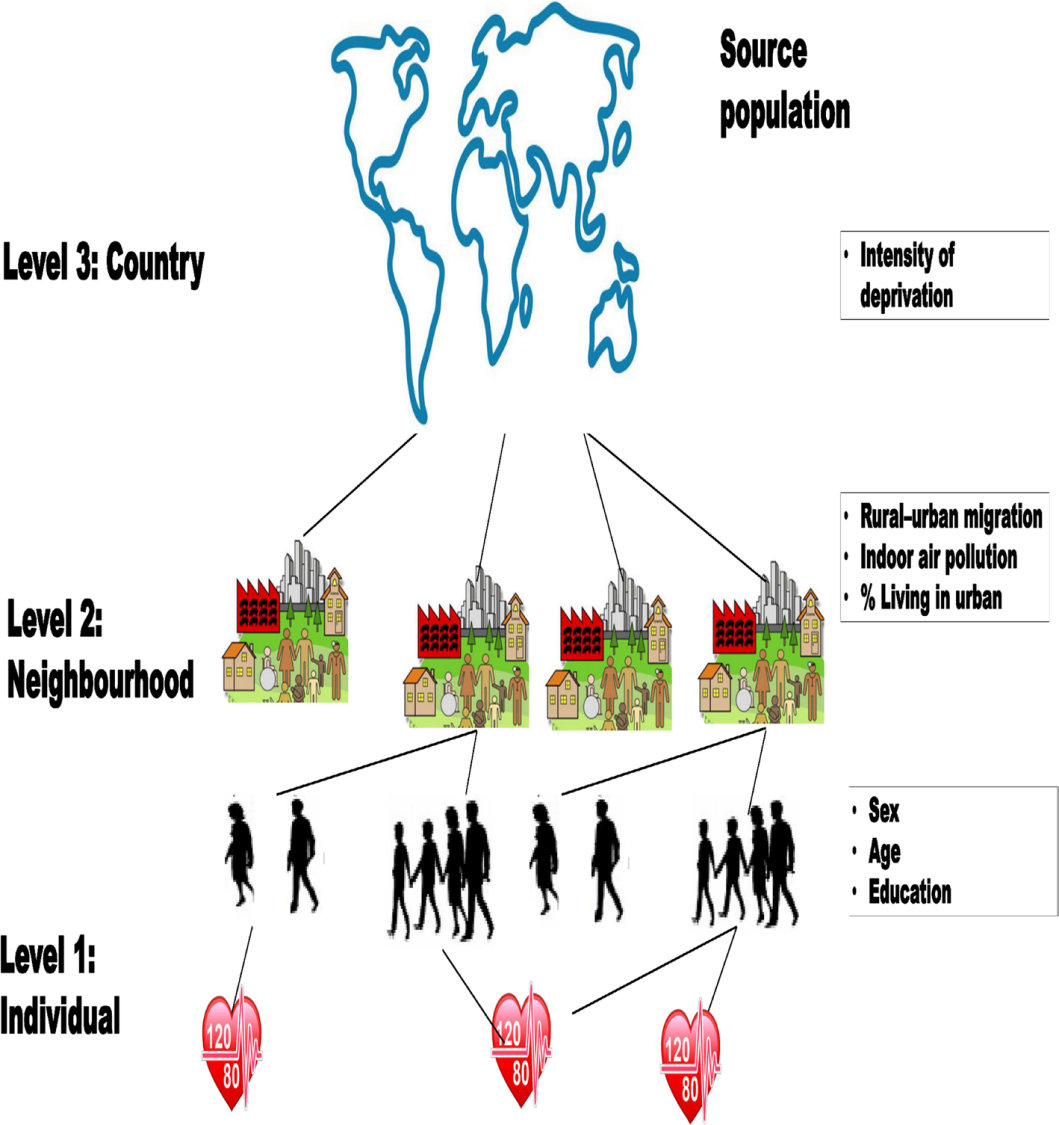
Multi-level data structure

The associations between individual compositional and contextual factors linked with hypertension were investigated using multivariable multi-level logistic regression models. For the binary hypertension risk, we built a multi-level logistic regression model for all respondents at level 1, respondents residing in a neighbourhood at level 2, and respondents living in a country at level 3 (see Fig. 1). We fitted the following five models: (1) To breakdown the variation across the country and neighbourhood levels, the first model was an empty or unconditional model without any explanatory variables; (2) The second model only included factors at the individual level; (3) The third model solely included neighbourhood-level variables; (4) The fourth model solely included country-level variables; and (5) The fifth model included factors at the individual, neighbourhood, and national levels at the same time (full model).

#### Fixed effects (measures of association)

The measures of association are reported as odds ratios (ORs) with their 95% credible intervals (CIs). We summarised the measures of association (ORs) with 95% credible intervals (95% CI) rather than 95% confidence intervals (95% CI) using Bayesian statistical inference, which provides probability distributions.

#### Random effects (measures of variation)

The intraclass correlation (ICC) and median odds ratio were used to assess the possible contextual effects (MOR) [34]. The ICC was used to determine the degree of similarity between respondents in the same neighbourhood and within the same country [34]. The ICC is a measure of clustering of the odds of hypertension in the same neighbourhood and nation, and it represents a proportion of the overall variance in the probability of hypertension linked to neighbourhood- and country-level factors. Snijders [48] used the linear threshold (latent variable approach) to calculate the ICC, and we reported neighbourhood impacts in terms of odds, as proposed by Snijders [48]. Larsen [28] The MOR calculates the chance of hypertension at the second or third level by calculating the variance in the neighbourhood or country as an odds ratio (neighbourhood or country context). When the MOR is 1 (one), there is no variation in the neighbourhood or country. When the MOR is higher, the contextual influences become more important in determining the risk of hypertension [34].

##### Model fit and specifications

The variance inflation factor (VIF) [50], all diagonal elements in the variance-covariance matrix for correlations between − 1 and 1, and diagonal elements for any elements close to zero were all examined for multi-collinearity among the explanatory variables. However, none of the test findings were cause for alarm because they were all within permissible limits. As a result, the models produce reliable and valid findings [50]. The software MLwinN, version 2.31, was used to analyse the data [6, 43].

## Results

### Sample characteristics

The list of countries, years of data collection, and survey characteristics are presented in Table 1. The median number of neighbourhoods sampled was 498, ranging from 316 in the Kyrgyz Republic to 28,408 in India. The number of respondents included in the analysis ranged from 3630 in Namibia to 781,117 in India. The prevalence of hypertension ranged from 10.3% in the Kyrgyz Republic to 52.2% in Haiti. The descriptive statistics for the final pooled sample are presented in Table 2. For this analysis, we analysed the data of 888,925 respondents (Level 1) nested within 33,883 neighbourhoods (Level 2) from 12 countries (Level 3) in LMICs. The majority of the respondents were female (84%), had secondary or higher education (61%), and were currently married (70%). One in five respondents did not have access to a newspaper, television or radio. A fairly equal number of respondents were exposed to indoor air pollution. Approximately 15% of the respondents had health insurance, and a few respondents reported that they had problems getting money needed for treatment. Only approximately 3% of the respondents reported a history of cigarette smoking. The percentage of individuals with hypertension was higher among males than females (21.2% versus 16.8%, *p* < 0.0001). Respondents from richer households had a significantly higher prevalence of hypertension than those from poorer households (20.4% versus 14.1%, *p* < 0.0001). Respondents who were overweight (30.0%) and obese (42.6%) were significantly more likely to be hypertensive than those who were normal weight (15.0%) and underweight (9.5%, *p* < 0.0001). The prevalence of current smoking was significantly higher among smokers than non-smokers (23.1% versus 16.9%, *p* < 0.0001).

**Table 1:** Description of Demographic and Health Surveys data in low- and middle-income countries, 2011 to 2018

Country	Survey year	Number of Neighbourhoods	Sample Size	Human Development Index	Hypertension (%)
Albania	2018	715	20,846	High	29.9
Bangladesh	2011	600	7887	Low	29.4
Benin	2018	555	6700	Low	22.2
Ghana	2014	427	13,741	Low	13.5
Haiti	2017	450	4615	Low	52.2
India	2015	28,408	781,117	Low	16.5
Kyrgyz Republic	2012	316	10,487	High	10.3
Lesotho	2014	399	6076	Low	19.5
Namibia	2013	546	3630	Low	46.2
Nepal	2016	383	14,823	Low	22.4
South Africa	2016	718	8346	High	48
Tajikistan	2017	366	10,657	High	12.1

**Table 2:** Summary of pooled sample characteristics of the Demographic and Health Surveys data in low- and middle-income countries, 2011 to 2018

			Normotensive	Hypertensive	
	Number	Percentage	Percentage	Percentage	
			%	%	*p*-value
Sex					0.000
Male	143,171	16.11	78.75	21.25	
Female	745,754	83.89	83.16	16.84	
Education					0.000
No education	216,545	24.41	80.37	19.63	
Primary	130,884	14.75	79.00	21.00	
Secondary+	539,716	60.84	84.12	15.88	
Wealth					0.000
Poorer	287,855	33.33	85.88	14.12	
Middle	287,852	33.33	82.74	17.26	
Richer	287,853	33.33	79.55	20.45	
Marital status					0.000
Never married	222,530	25.87	92.32	7.68	
Currently married	603,679	70.17	79.83	20.17	
Previously married	34,129	3.97	74.66	25.34	
BMI					0.000
Underweight	178,086	20.42	90.46	9.54	
Normal weight	518,977	59.49	84.97	15.03	
Overweight	131,873	15.12	70.01	29.99	
Obese	43,372	4.97	57.45	42.55	
Access to media					0.000
0	202,765	22.81	85.45	14.55	
1	325,536	36.62	82.01	17.99	
2	305,967	34.42	81.71	18.29	
3	54,657	6.15	77.96	22.04	
Indoor air pollution					0.000
Low	444,874	50.05	80.92	19.08	
High	444,049	49.95	83.98	16.02	
Have any health insurance					0.000
No	754,999	84.93	82.80	17.20	
Yes	133,926	15.07	81.30	18.70	
Problem getting money needed for treatment					0.000
No	534,161	73.65	83.60	16.40	
Yes	191,145	26.35	82.82	17.18	
Cigarette smoking					0.000
No	821,951	97.09	83.10	16.90	
Yes	24,669	2.91	76.92	23.08	
Neighbourhood disadvantage					0.000
Least	296,341	33.34	79.47	20.53	
Moderate	296,308	33.33	82.06	17.94	
Higher	296,276	33.33	85.81	14.19	
Human development index					0.000
Low	396,577	44.61	82.08	17.92	
Moderate	442,012	49.72	83.62	16.38	
High	50,336	5.66	74.96	25.04	

### Measures of associations (fixed effects)

The results of the different models are shown in Table 3. In the fully adjusted model, we controlled for the effects of the individual-, neighbourhood- and country-level factors and our findings show that for every 10-year increase in the age of the respondents, the odds of developing hypertension increased by 74% (OR = 1.74, 95% CI 1.73 to 1.76). The odds of developing hypertension increased with increasing educational attainment and wealth index. Respondents with secondary or higher education were 4% more likely to develop hypertension than those with no education (OR = 1.04, 95% CI 1.02 to 1.06). Similarly, respondents from wealthier households were 8% more likely to develop hypertension (OR = 1.08, 95% CI 1.05 to 1.12). Respondents who were currently (OR = 1.35, 95% CI 1.32 to 1.38) or ever married (OR = 1.37, 95% CI 1.32 to 1.42) were 35 and 37% more likely to have hypertension than those who were never married, respectively. Respondents who were overweight (OR = 1.72, 95% CI 1.69 to 1.75) and obese (OR = 2.67, 95% CI 2.60 to 2.74) were almost two and three times more likely to have hypertension than those with normal body weight. Respondents who reported money problems with regard to assessing care were 8% more likely to have hypertension (OR = 1.08, 95% CI 1.06 to 1.09) than those who do not have money problems. Respondents who smoked cigarettes were 13% more likely to have hypertension than those who did not smoke cigarettes (OR = 1.13, 95% CI 1.04 to 1.23). Respondents living in the least deprived areas were 14% more likely to have hypertension than those from the most deprived areas (OR = 1.14, 95% CI 1.10 to 1.17).

**Table 3:** Individual compositional and contextual factors associated with hypertension identified by multivariable multi-level logistic regression models, Demographic and Health Surveys data, 2011–2018

	Model 1^**a**^		Model 2^**b**^		Model 3^**c**^		Model 4^**d**^		Model 5^**e**^	
	OR (95% CI)		OR (95% CI)		OR (95% CrI)		OR (95% CI)		OR (95% CI)	
**Fixed-effect**										
**Individual-level factors**										
Male (vs female)					0.95^***^	[0.94,0.97]	0.95^***^	[0.93,0.97]		
Age per 10 years			1.74^***^	[1.73,1.76]	1.88^***^	[1.87,1.89]	1.90^***^	[1.89,1.91]	1.74^***^	[1.73,1.76]
Education attainment										
No education										
Primary			1.09^***^	[1.06,1.11]					1.08^***^	[1.05,1.10]
Secondary or higher			1.05^***^	[1.03,1.07]					1.04^***^	[1.02,1.06]
Wealth index										
Poorer										
Middle			1.10^***^	[1.08,1.12]					1.08^***^	[1.05,1.10]
Richer			1.11^***^	[1.08,1.14]					1.08^***^	[1.05,1.12]
Marital status										
Never married										
Currently married			1.35^***^	[1.32,1.38]					1.35^***^	[1.32,1.38]
Ever married			1.37^***^	[1.32,1.42]					1.37^***^	[1.32,1.42]
Body mass index										
Underweight			0.79^***^	[0.77,0.80]					0.79^***^	[0.77,0.80]
Normal weight										
Overweight			1.72^***^	[1.69,1.75]					1.72^***^	[1.69,1.75]
Obese			2.67^***^	[2.60,2.74]					2.67^***^	[2.60,2.74]
Media access			1.02^**^	[1.00,1.03]					1.01^*^	[1.00,1.02]
Indoor air pollution			0.99	[0.97,1.01]					0.99	[0.97,1.01]
Health insurance			1.00	[0.99,1.02]					1.00	[0.98,1.02]
Money problem			1.08^***^	[1.06,1.10]					1.08^***^	[1.06,1.09]
Cigarette smoker			1.14^**^	[1.05,1.24]					1.13^**^	[1.04,1.23]
**Neighbourhood-level factors**										
**Neighbourhood disadvantage**										
Least										
Moderate					1.47^***^	[1.44,1.51]			1.10^***^	[1.06,1.14]
Higher					1.32^***^	[1.30,1.36]			1.14^***^	[1.10,1.17]
**Country-level factors**										
**Human development index**										
**Low**										
Moderate							0.77	[0.44,1.34]	1.34	[0.63,2.85]
High							0.54	[0.24,1.21]	0.96	[0.37,2.51]
**Random effects**										
Country-level										
Variance (95 CrI)	1.65^*^	[1.11,2.48]	1.20^*^	[1.02,1.41]	1.16^*^	[1.03,1.30]	1.13^*^	[1.02,1.24]	1.19^*^	[1.02,1.38]
VPC (%, 95 CrI)	26.3	[19.40,34.80]	18.8	[16.50,21.30]	19.3	[17.60,21.10]	17.5	[16.20,18.80]	18	[15.90,20.20]
MOR (95% CrI)	3.41	[2.73,4.49]	2.84	[2.62,3.10]	2.79	[2.63,2.97]	2.76	[2.62,2.89]	2.83	[2.62,3.07]
Explained variation (%)		[.,.]	27.3	[8.10,43.10]	29.7	[7.20,47.60]	31.5	[8.10,50.00]	27.9	[8.10,44.40]
Neighbourhood-level										
Variance (95 CrI)	1.34^***^	[1.33,1.35]	1.90^***^	[1.87,1.93]	1.56^***^	[1.54,1.57]	2.03^***^	[2.00,2.06]	2.14^***^	[2.10,2.17]
VPC (%, 95 CrI)	47.6	[42.60,53.80]	48.5	[46.70,50.40]	45.2	[43.80,46.60]	49	[47.80,50.10]	50.3	[48.70,51.90]
MOR (95% CrI)	3.02	[3.00,3.03]	3.72	[3.69,3.76]	3.29	[3.27,3.30]	3.89	[3.85,3.93]	4.04	[3.98,4.08]
Explained variation (%)		[.,.]	−41.8	[−40.60,-43.00]	−16.4	[−15.80,-16.30]	−51.5	[−50.40,-52.60]	−59.7	[−57.90,-60.70]

^a^Model 1 – empty null model, baseline model without any explanatory variables (unconditional model)

^b^Model 2 – adjusted for only individual-level factors

^c^Model 3 – adjusted for only neighbourhood-level factors

^d^Model 4 – adjusted for only country-level factors

^e^Model 5 – adjusted for individual-, neighbourhood-, and country-level factors (full model)

*OR* odds ratio, *CI* confidence interval, *MOR* median odds ratio, *VPC* variance partition coefficient

^*^ *p* < 0.05, ^**^ *p* < 0.01, ^***^ *p* < 0.001

### Measures of variations (random effects)

We observed a significant variation in the odds of hypertension across the countries (*σ*^2^= 1.65, 95% CI 1.11 to 2.48) and across the neighbourhoods (*σ*^2^= 1.34, 95% CI 1.33 to 1.35). Table 3 shows the results for Model 1 (unconditional model). The intra-country and intra-neighbourhood correlation coefficients indicate that 26.3 and 47.6% of the variance in the odds of hypertension could be attributed to country- and neighbourhood-level factors, respectively. The median odds ratio (MOR) results also confirmed that neighbourhood and societal contextual phenomena influence the individual risk of developing hypertension. The results from the full model (Model 5) show that if a respondent moved to another country or another neighbourhood with a higher probability of hypertension, the median increase in their odds of hypertension would be 2.83-fold (95% CI 2.62 to 3.07) and 4.04-fold (95% CI 3.98 to 4.08), respectively.

## Discussion

To our knowledge, the current study is the first multi-level examination of hypertension risk in low- and middle-income countries. Like most traditional epidemiological studies that had examined traditional well-established individual-level risk factors, we found that increasing age, educational attainment, wealth status, overweight/ obesity, and cigarette smoking were associated with increased risk of hypertension. The findings revealed that differences between neighbourhoods and countries determine hypertension risk. The results show that the prevalence of hypertension is still relatively high in LMICs. Previous studies by Kibria et al. [24] & Chowdhury et al. [10] in cross-sectional studies revealed high hypertension prevalence in Bangladesh, Nepal, and other LMICs. The increase in the prevalence of hypertension had been linked to rapid urbanisation and unhealthy lifestyle changes, such as the consumption of unhealthy diets that include fast foods, sedentary behaviour and increased alcohol consumption [51].

After adjusting and controlling for the effects of the individual-, neighbourhood- and country-level factors, we observed that for every 10-year increase in age, there was an increase in the odds of developing hypertension. Our findings were consistent with Chowdhury et al. [10] & Hassan et al. [19]. Both studies found that the prevalence of hypertension increases with increasing age and being affected by the place of residence, sex, education, wealth index, working status and body mass index. Ageing increases the risk of infection and diseases, which in turn elevate the risk of mortality [8].

The odds of developing hypertension increased with increasing levels of educational attainment and wealth index values. Respondents with secondary or higher education levels were more likely to develop hypertension than those with no education. Similarly, respondents from wealthier households were more likely to develop hypertension. Supporting this finding, Kibria et al. [24], Tareques [49] & Sanuade et al. [46] noted that the prevalence of hypertension was higher in urban individuals and those in higher socioeconomic classes (i.e., the highest wealth quintile) than in respondents from rural areas and the lowest wealth quintile. The odds of developing hypertension in higher socioeconomic classes and urban individuals can be attributed to several behavioural factors, such as unhealthy diet, cigarette use, and alcohol consumption are influenced by place of residence, wealth status and level of education [55]. To address these risk factors, behaviour change programmes should be tailored to wealthier populations and individuals with higher educational attainment to reduce the incidence of hypertension in this group.

Our findings also revealed that Marital status is a significant independent predictor of hypertension. We found that currently married or ever married respondents had an increased chance of developing hypertension compared to respondents who reported never being married in LMICs. Supporting this evidence, Tuoyire & Ayetey [51] & Sanuade et al. [46] both studies established that being currently married or previously been married status increased the odds of developing hypertension in Ghanaian women. This could result from low income or inability to access health care facilities and much stress from daily struggle.

The association between weight gain and blood pressure has been well studied. We found that individuals who were overweight and obese were almost two and three times more likely to develop hypertension than those with normal body weight. Our result is consistent with studies carried out by Harshfield et al. [18], Rahman et al. [42], Taraque et al. [49], Chowdhoury et al. [10], Alkibria et al. [1] & Fottrell et al. [15]. These studies confirmed that being obese or overweight is a traditional risk factor for hypertension. Rahman and colleagues estimated the prevalence of hypertension to be higher in urban areas than in rural areas. People living in urban areas may consume more calories and have sedentary lifestyle patterns that may increase BMI, thereby increasing their risk of developing hypertension.

We noted that individuals who reported financial problems assessing health care were at higher risk of developing hypertension than those financially stable. The lack of universal health insurance coverage has been a significant barrier to accessing health care facilities in most LMICs [46]. The rapid increase in the prevalence of hypertension is due to low and current projected spending on health. Despite the high number of populations in these regions, only 0.4% of global health spending was in LMICs in 2016 [35].

The results of this study indicate that smoking is a vital determining factor with regard to developing hypertension. We observed that respondents who smoked cigarettes were more likely to have hypertension than those who did not smoke. This finding is consistent with that of Saladini et al. [45]. The study revealed that the odds of developing hypertension are elevated in those who smoke. Tobacco smoking is associated with increased arterial wall stiffness, thereby increasing the risk of developing hypertension [2].

The study also found that the place of residence plays a significant role in determining the health outcome of individuals. Respondents living in the least deprived areas were more likely to develop hypertension than those in the most deprived areas. Chowdhury et al. [10], Kibra et al. [24] & Sanuade et al. [46] noted that place of residents had significant associations with hypertension in rural and urban regions among older people, wealthier people, females, those with diabetes and overweight individuals. Based on the distributions of these significant factors, it is highly likely that public health awareness campaigns targeted at deprived areas could contribute to controlling hypertension globally.

More crucially, the findings add to the body of knowledge by revealing those factors at the contextual level increase hypertension risk in addition to individual-level determinants. Researchers have recently become more interested in exploring the effects of contextual SES on CVD risk variables [11, 13]. Several studies have found that neighbourhood SES traits are inversely related to blood pressure reactivity, suggesting that individual and neighbourhood SES may be independent predictors of blood pressure [22, 29, 30, 33]. According to Matheson et al. [32], deprivation in the neighbourhood appears to be a stronger predictor of hypertension in women. Women living in high deprivation areas are 10% more likely to report having hypertension than males living in the same areas and women living in the least deprived areas. Liu and colleagues also discovered that variations in the prevalence of high blood pressure can account for between 44 and 53% of the variation in the prevalence of high blood pressure and that individuals living in disadvantaged physical and socioeconomic environments have a significantly higher risk of high blood pressure prevalence [30].

We found evidence of geographical clustering in the risk of hypertension. Differences between countries and neighbourhoods accounted for approximately 26 and 48% of the variation in hypertension, respectively. We also observed that respondents moving to a different neighbourhood or country with a higher risk of hypertension had an increased chance of developing hypertension. People from the same community are inherently more similar in terms of their current risk of developing hypertension than people from different neighbourhoods, i.e., the contextual phenomenon manifests itself as the clustering of the risk of hypertension within neighbourhoods. Researchers frequently use an ecological perspective to understand the risk of developing a disease [3–5]. The disease is viewed as a multidimensional phenomenon including the interaction of individual, family, community, and societal factors88 in this paradigm. The framework considers the many levels of societal organisation as well as their impact on disease. An individual lives in a household unit, which is part of a community that is governed by the policies of a state or national government. Every level of the social hierarchy can impact an individual’s illness risk. The ecological model also promotes a complete public health strategy that tackles not only an individual’s risk of becoming a hypertension risk.

Based on these contextual findings, we might infer that there is some evidence for a possible neighbourhood and country contextual phenomenon shaping a common individual hypertension risk. These results highlight the importance of implementing public health preventive measures at the high-risk person level and at the high-risk neighbourhood level. Interventions that target an individual’s social and physical settings and health care systems are required to eliminate or reduce hypertension risk. These interventions must be multidimensional, i.e., they must take place at multiple levels simultaneously or in close succession [40]. Changes in behaviour, such as eating better foods; legislative changes, such as raising taxes on unhealthy foods or drinks; changes in the delivery of health services; and environmental changes, such as making healthy food more accessible, are all possible outcomes of multi-level interventions [40]. Community-based programmes, which connect communities and health systems and involve a variety of treatments like education and outreach, self-management, and home-based care, have emerged as a viable way to close access gaps [7, 31, 41] According to previous studies, community-based hypertension screening and case management strategies can save money while also improving outcomes [21, 25].

Further research is needed to construct novel risk scores for hypertension that include both individual and contextual factors to identify people at higher risk for developing hypertension and examine opportunities for the improved use of preventive interventions and the targeted delivery of proactive, personalised treatment. The current methods do not account for the underlying contextual factors that contribute to hypertension. There are significant health and economic benefits to early diagnosis, proper care, and effective hypertension control. Treating complications necessitates expensive measures that deplete the budgets of both individuals and governments. Similarly, additional decomposition studies may provide more information about important factors that could explain the differences in the risk of hypertension among high-risk individuals and those living in high-risk locations.

Some study limitations should be considered when interpreting our findings. First, we could not measure the length of time participants had lived in their current neighbourhoods and the degree of their exposure to the environment. Thus, we could not determine whether the associations of neighbourhood characteristics with current hypertension were due to cumulative effects. Second, the data we used were cross-sectional; hence, we were unable to determine causal inferences with regard to the reported associations. Finally, DHS surveys do not collect information on household expenditures or incomes, which are established indicators used to measure wealth. Montgomery et al. [36] & Filmer [14] reported that the asset-based wealth index could only be used as a proxy indicator of household economic status. The results obtained from the direct measurement of income and expenditures where such data are available are more reliable and consistent. The number of participants from India included in the analysis was considerably greater than the numbers from other countries; therefore, the characteristics of those respondents may have influenced the risk factors identified.

Despite these limitations, the findings of our study are significant because the data used are from a large, population-based survey with high response rates covering 12 LMICs. The DHS is a nationally representative survey that allows the drawing of conclusions across countries. The DHS data were collected using the same approach in all participating countries, allowing comparisons across countries.

In conclusion, socioeconomic position with regard to individual, compositional and contextual measures was independently associated with the risk of developing hypertension. Based on these findings, it is highly likely that these countries will benefit immensely from multi-level hypertension prevention strategies that address the different contextual risk factors explored in the study. Further studies could explore how these strategies could help reduce the incidence of hypertension and other related comorbidities, such as diabetes, coronary heart failure, obesity, etc., in the general population.

## Data Availability

All methods were carried out in accordance with relevant guidelines and regulations, and data were sourced from the link below. The data supporting this article are available at: http://dhsprogram.com/data/available-datasets.cfm.
